# Genetic, DNA methylation, and immune profile discrepancies between early-stage single primary lung cancer and synchronous multiple primary lung cancer

**DOI:** 10.1186/s13148-023-01422-y

**Published:** 2023-01-07

**Authors:** Fenglei Yu, Xiaojie Huang, Danting Zhou, Zhenyu Zhao, Fang Wu, Banglun Qian, Qiang Wang, Juan Chen, Qingchun Liang, Yi Jiang, Qi Ding, Qiongzhi He, Jingqun Tang, Xiang Wang, Wenliang Liu, Chen Chen

**Affiliations:** 1 Department of Thoracic Surgery, Second Xiangya Hospital of Central South University, Changsha, 410011 Hunan People’s Republic of China; 2 Department of Cardiovascular Surgery, Second Xiangya Hospital of Central South University, Changsha, Hunan People’s Republic of China; 3 Department of Oncology, Second Xiangya Hospital of Central South University, Changsha, Hunan People’s Republic of China; 4 Department of Radiology, Second Xiangya Hospital of Central South University, Changsha, Hunan People’s Republic of China; 5 Department of Pathology, Second Xiangya Hospital of Central South University, Changsha, Hunan People’s Republic of China; 6 Hunan Key Laboratory of Early Diagnosis and Precise Treatment of Lung Cancer, Second Xiangya Hospital of Central South University, Changsha, People’s Republic of China; 7 Geneplus-Beijing Institute, Beijing, People’s Republic of China

**Keywords:** Synchronous multiple primary lung cancers, Gene mutation, DNA methylation, Immune profile, Tumor microenvironment

## Abstract

**Background:**

To explore the possible carcinogenesis and help better diagnose and treat patients with synchronous multiple primary lung cancers (sMPLC), we systematically investigated the genetic and DNA methylation profiles of early-stage sMPLC and single primary lung cancer (SPLC) and explored the immune profiles in the tumor microenvironment.

**Methods:**

Hundred and ninety-one patients with 191 nodules in the SPLC group and 132 patients with 295 nodules in the sMPLC group were enrolled. All the samples were subjected to wide panel-genomic sequencing. Genome-wide DNA methylation was assessed using the Infinium Human Methylation 850 K BeadChip. RNA-seq and CIBERSORT analyses were performed to identify the immune characteristics in these two groups.

**Results:**

Lesions from sMPLC patients had lower TMB levels than that from SPLC patients. sMPLC had a similar genetic mutational landscape with SPLC, despite some subgroup genetic discrepancies. Distinct DNA methylation patterns were identified between the two groups. The differentially methylated genes were related to immune response pathways. RNA-seq analyses revealed more immune-related DEGs in sMPLC. Accordingly, more immune-related biological processes and pathways were identified in sMPLC. Aberrant DNA methylation was associated with the abnormal expression of immune-related genes. CIBERSORT analysis revealed the infiltration of immune cells was different between the two groups.

**Conclusion:**

Our study for the first time demonstrated genetic, epigenetic, and immune profile discrepancies between sMPLC and SPLC. Relative to the similar genetic mutational landscape, the DNA methylation patterns and related immune profiles were significantly different between sMPLC and SPLC, indicating their essential roles in the initiation and development of sMPLC.

**Supplementary Information:**

The online version contains supplementary material available at 10.1186/s13148-023-01422-y.

## Background

With the widespread use of high-resolution computed tomography (CT) for lung cancer screening, patients with synchronous multiple primary lung cancers (sMPLC) are becoming a rapidly growing population in clinical practice [1]. The crucial issue is whether these coexisting lesions should be diagnosed and treated as separate primary lesions or metastasis, as the prognosis and treatment strategies vary between the two forms of the disease [2, 3]. The current diagnosis of sMPLC uses the criteria defined by Martini and Melamed [4] (updated in 2007 [5]), which is mainly based on the clinical and histological features of the lesions. It has become increasingly accepted for conceptually understanding the nature and the clonality of the lesions [6, 7]. Undoubtedly, a more precise interpretation of the clonal origin and carcinogenesis of sMPLC will facilitate the treatment and improve patient survival [8–10].

Previous studies have addressed that the *EGFR, KRAS, BRAF,* and *ERBB2* genes have a higher rate of mutational frequency in patients with sMPLC, indicating that histologic and genetic evaluation may be an optional diagnostic strategy for the identification and treatment of sMPLC [7, 10, 11]. In addition, these mutations are of significant interest in the study of lung adenocarcinomas, as they are known to be ‘early events’ in carcinogenesis [12]. Moreover, it has been revealed that the tumor lesion is a complex ecosystem composed of malignant cells, various types of immune cells, and stromal cells [13]. The heterogeneity of tumor cells and different types of tumor-infiltrating lymphocytes (TILs) played essential roles in tumor behaviors [14–16]. A certain group of infiltrating immune cells, such as CD8^+^ T cells, regulatory T cells (Tregs), and myeloid-derived suppressor cells, have been identified to be critical factors in the progression of malignant tumors [17–19]. However, previous studies mainly focused on the genetic changes and the heterogeneity of sMPLC. There remains a lack of deep insight into the molecular events and immune response in the occurrence and progression of sMPLC. To address this gap in our knowledge, we systematically investigated the genomic profiles and DNA methylation patterns of early-stage single primary lung cancer (SPLC) and sMPLC and explored the immune profiles in the tumor microenvironment. By comparing the genetic, epigenetic, and immune landscapes between sMPLC and SPLC, our study provides new insights into the biology and possible carcinogenesis of sMPLC, with implications for disease monitoring and future therapeutic interventions.

## Results

Among the 337 patients, 7 patients from the SPLC group were pathologically diagnosed with squamous cell carcinoma (*n* = 5) and large-cell neuroendocrine carcinoma (*n* = 2), and 7 patients were found to have lymph node metastasis at the time of surgery (3 patients in SPLC group and 4 patients in sMPLC group). These 14 patients were excluded from the following analyses. In all, it included 191 patients with 191 lung nodules in the SPLC group and 132 patients with 295 lung nodules in the sMPLC group. All lesions were no larger than 3.0 cm in the greatest dimension verified by pathological reports. Nodules from sMPLC patients showed significant genetic heterogeneity. Most patients with multifocal adenocarcinomas were histopathologically different or harbored different genetic alternations. Nevertheless, about 8.3% (11/132) of the patients in the sMPLC group had the same gene mutation profiles. CT features and histopathological analyses were re-evaluated for these patients to confirm the sMPLC diagnosis [5, 9]. Based on CT findings, pathological results, and follow-up data, all 323 patients were diagnosed as T_1-2_N_0_M_0_ (stage Ia-Ib) SPLC or sMPLC patients. (Some tumors were T2 based on visceral pleural invasion and not size.) Clinical and demographic variables are shown in Additional file 1: Table S1.

### The tumor mutation burden in SPLC and sMPLC patients

**Fig. 1:**
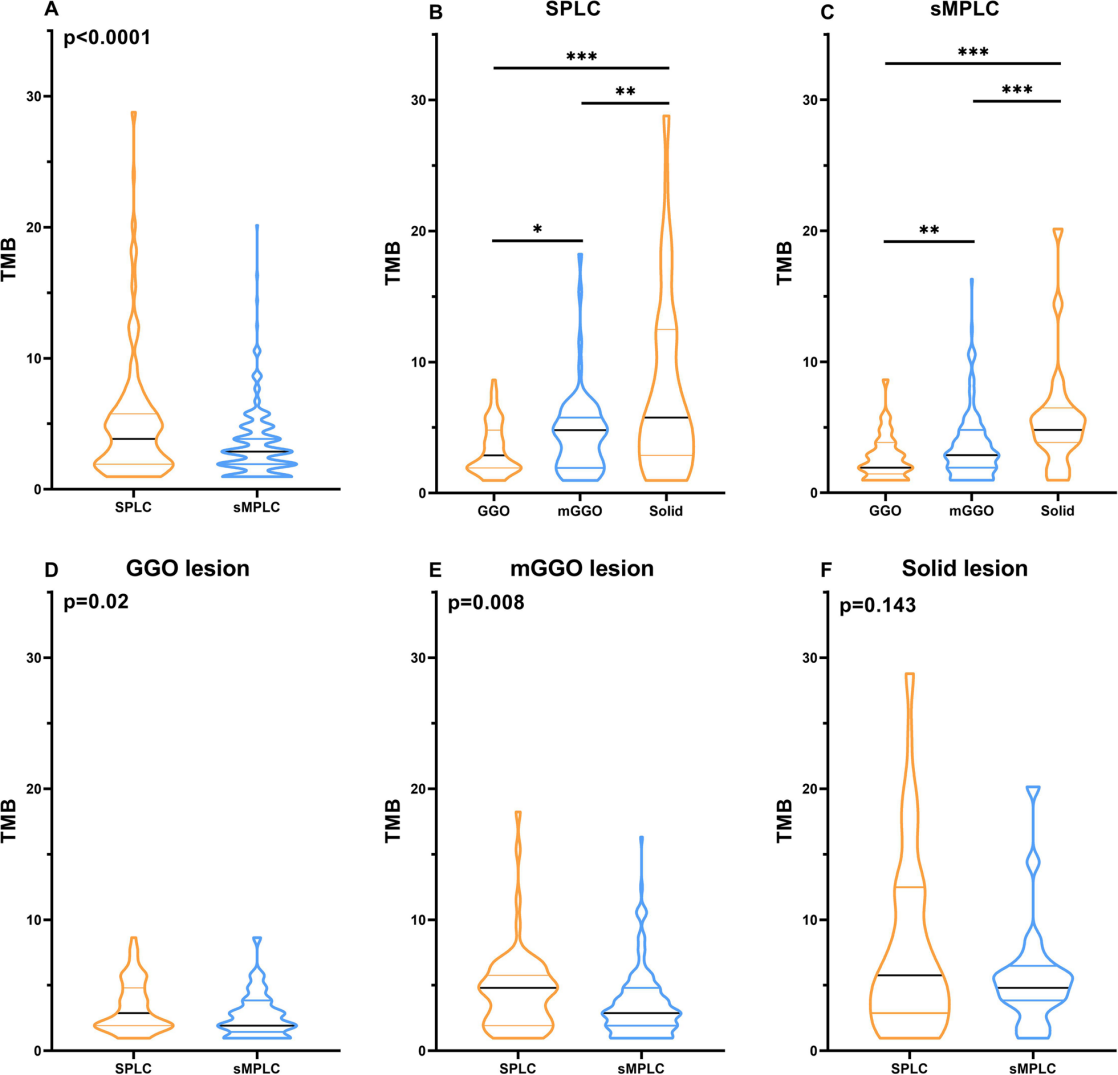
Tumor mutation burden in SPLC and sMPLC patients. **A** Generally, sMPLC lesions had lower TMB levels than SPLC nodules. **B** and **C** in both SPLC and sMPLC patients, TMB gradually increased from GGO lesions to mGGO and solid lesions. **D** and **E** When compared between the two groups, the TMB of GGO and mGGO lesions from sMPLC patients was significantly lower than that of SPLC patients. **F** The solid nodules from sMPLC patients also showed a similar trend, but no statistical significance was found. *, *p* < 0.05; **, *p* < 0.01; ***, *p* < 0.001

We first investigated the tumor mutation burden (TMB) status. The changes in TMB showed similar trends in both groups. TMB was higher in older patients and gradually increased parallel to the tumor size (Additional file 2: Table S2). In terms of CT features, TMB gradually increased from ground-glass opacity (GGO) lesions to mixed GGO (mGGO) and solid lesions (Fig. 1B and C), while in terms of pathological subtypes, TMB was significantly higher in invasive adenocarcinoma (IAC) lesions than that in adenocarcinoma in situ (AIS)/minimally invasive adenocarcinoma (MIA) lesions in both groups (Additional file 2: Table S2).

We then investigated the TMB differences between the two groups. In terms of patient age, TMB of sMPLC lesions showed a lower trend than SPLC lesions in all age-groups, despite statistical difference only being found in the subgroup of 40- to 60-year-old patients (*p* < 0.0001). Although the TMB was similar between the two groups in the nodules less than 1 cm, patients from the SPLC group had significantly higher TMB levels when the tumor size was larger than 1 cm (Additional file 2: Table S2). Interestingly, when compared between the two groups, the TMB of GGO and mGGO lesions from sMPLC patients was significantly lower than that of SPLC patients (Fig. 1D and E, Additional file 2: Table S2). The solid nodules from sMPLC patients also showed a similar trend, but no statistical significance was found (Fig. 1F). The TMB had no differences in AIS/MIA lesions between the two groups, but in IAC nodules, sMPLC patients had significantly lower TMB levels than SPLC patients. Our study demonstrated that sMPLC lesions had lower TMB levels than those from SPLC nodules (Fig. 1A, Additional file 2: Table S2).

### Gene mutation landscape in SPLC and sMPLC patients

Gene mutational analyses were performed on all the lesions included in this study. In both groups, a higher frequency of *EGFR* mutation was found in females (69.4% in the SPLC group and 63.0% in the sMPLC group), while *KRAS, LRP1B, FAT1, GRIN2A, KEAP1, STK11, TP53, PIK3CG, RET*, and *RB1* were more mutated in males (Additional file 3: Table S3A and F). The *MED12* and *FGFR3* mutations were more detected in younger patients, and *ARID1A* was more mutated in older cases (Additional file 3: Table S3B). In the sMPLC group, the *ERBB2, NOTCH4, KCNQ2*, and *XRCC3* mutations were more found in younger patients and mutated *RBM10, TP53, LRP1B, PTPRD*, and *STK11* were more detected in the patients more than 60 years old (Additional file 3: Table S3G). Similar to previous studies in both groups, the gene mutation rate significantly increased as the tumor became larger, except for *ERBB2*, which was more mutated in smaller lesions (Additional file 3: Table S3C and H). When comparing the gene mutation among GGO, mGGO, and solid lesions, the *EGFR* mutation was detected in the SLPC group (61.7% of GGO lesions, 70.0% of mGGO lesions, and 41.3% of solid nodules) and in the sMPLC group (44.8% of GGO lesions, 65.8% of mGGO lesions, and 79.2% of solid nodules). The mutations of *RET, PIK3CG*, and *PDGFRA* were only found in solid nodules. In the SPLC group, the mutation frequency of *TP53, KRAS, PTPRD, KEAP1, SMAD4, STK11, RB1*, and *GRIN2A* became higher as the consolidation part of the lesion increased (Additional file 3: Table S3D). The mutation rate of T*P53, MAP3K1, IFNG, MYC, CTNNB1, PIK3CG, MDM2*, and *NOTCH2* significantly increased from GGO lesions to solid lesions in sMPLC patients (Additional file 3: Table S3I). The *TP53* gene mutation was the most enriched alteration in the IAC compared to AIS/MIA lesions. The *MED12, BRCA1*, and *SOX17* mutations were found more in AIS/MIA lesions (Additional file 3: Table S3E). When investigating the significantly mutated genes according to histological features in the sMPLC group, the mutation rate of *TP53* was significantly higher in IAC lesions, and the *MAP2K1, BCOR, MED12, MST1R,* and *BRAF* mutations were more enriched in MIA/AIS nodules (Additional file 3: Table S3J).

### Genetical discrepancies between SPLC and sMPLC patients

**Fig. 2:**
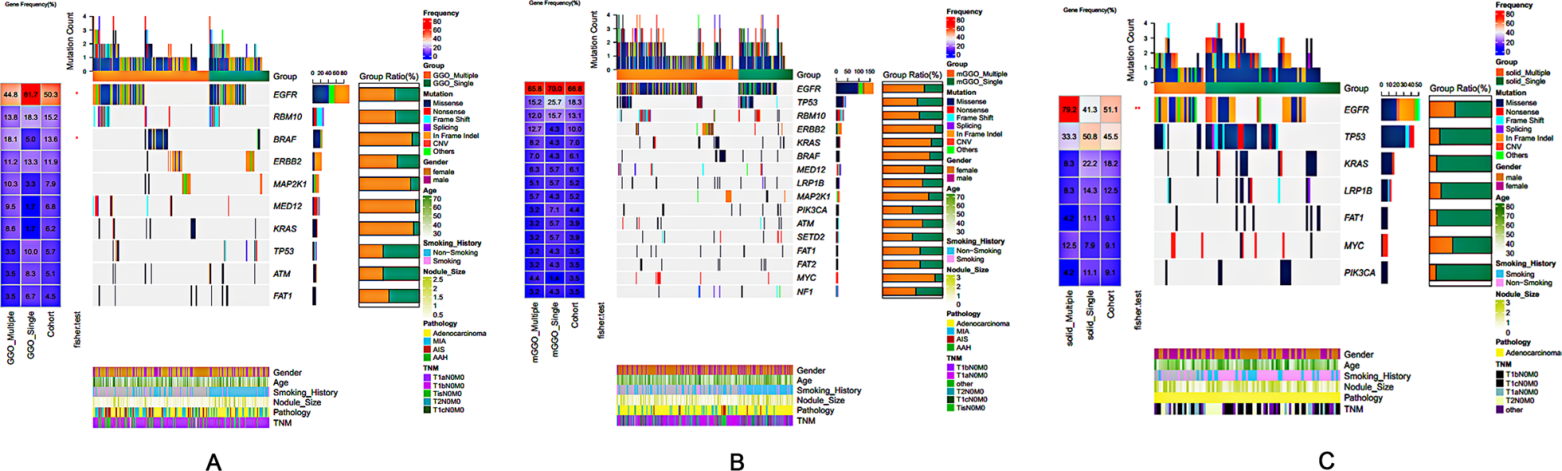
Genetical discrepancies between SPLC and sMPLC patients-CT feature oriented. **A** In GGO lesions, the EGFR mutation rate was lower in the sMPLC group than that in the SPLC group (44.8% vs. 61.7%, *p* < 0.05), while BRAF mutation was more frequently observed in sMPLC cases (18.1% vs. 5.0%, *p* < 0.05). **B** Gene mutation status had no differences between the two groups in mGGO lesions. **C** In solid lesions, the EGFR mutation was significantly higher in the sMPLC group than in SPLC patients (79.2% vs. 41.3%, *p* < 0.01)

The gene mutational discrepancies were investigated between SPLC and sMPLC patients. In GGO lesions, the *EGFR* mutation rate was lower in the sMPLC group than that in the SPLC group (44.8% vs. 61.7%, *p* < 0.05, Fig. 2A), while *BRAF* mutation was more frequently observed in sMPLC cases (Fig. 2A). Gene mutation status had no differences between the two groups in mGGO lesions (Fig. 2B). Interestingly, the *EGFR* mutation was significantly higher in solid lesions from the sMPLC group (79.2% vs. 41.3%, *p* < 0.01, Fig. 2C), which was the opposite of the GGO lesions.

We further analyzed the gene mutation differences in subgroups regarding gender, age, nodule size, and CT features. The gene mutation rate did not differ between the two groups in nodule size. In female patients with GGO lesions, *BRAF* mutation was more found in the sMPLC group (16.3% vs. 2.4%, *p* = 0.033), while *APC* (0.0% vs. 7.1%, *p* = 0.036) and *EGFR* (47.5% vs. 67.0%, *p* = 0.037) mutation rate was higher in SPLC group (Additional file 4: Table S4). Most patients in this study were between 40 and 60 years old. In this age period, the patients with multiple GGO lesions harbored more *BRAF* (18.6% vs. 2.5%, *p* = 0.022) mutation and less *APC* (0.0% vs. 10.0%, *p* < 0.01) mutation (Additional file 5: Table S5B). A higher frequency of *TP53* mutation of mGGO lesions was found in SPLC patients (30.0% vs. 13.0%, *p* = 0.028, Additional file 5: Table S5E). In the patients with solid nodules, *EGFR* mutation was significantly enriched in the sMPLC group (81.0% vs. 38.0%, *p* = 0.0067, Additional file 5: Table S5H).

### Overall changes in DNA methylation patterns in sMPLC and SPLC

**Fig. 3:**
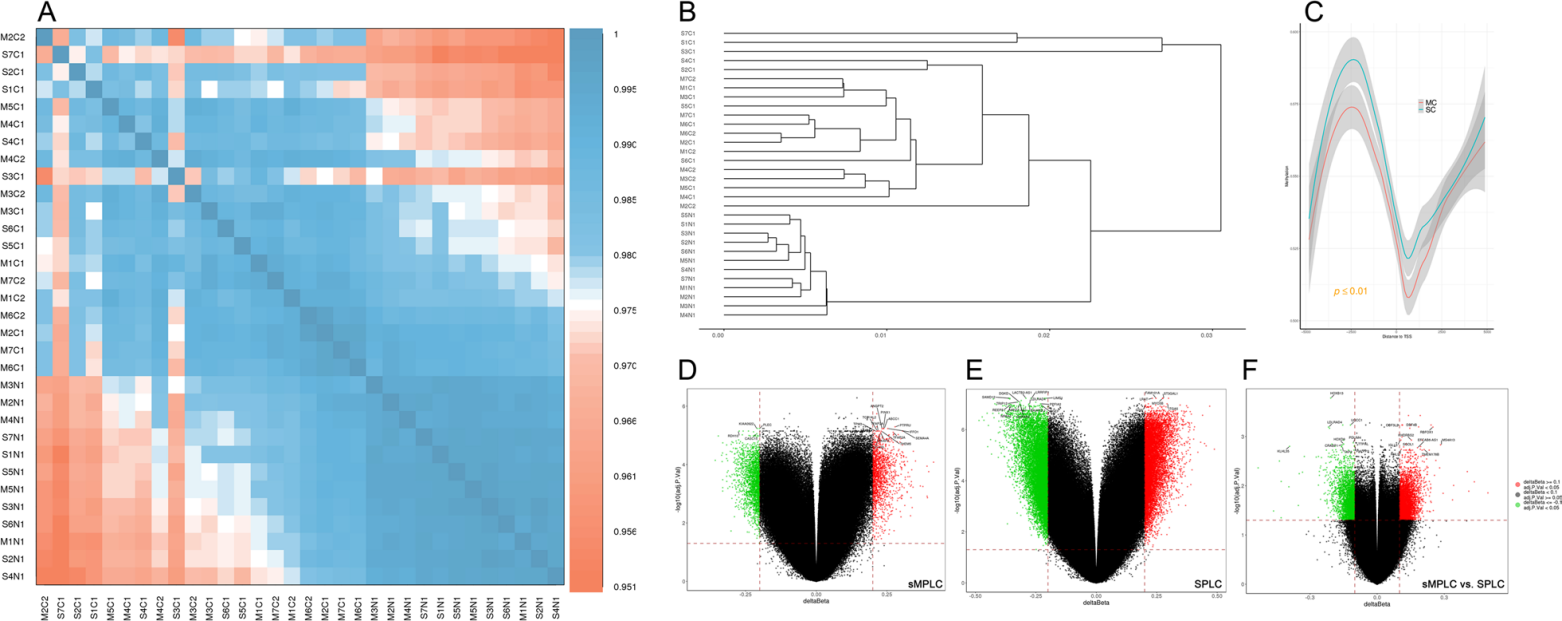
Genome-wide DNA methylation patterns in sMPLC and SPLC. **A** The heatmap and cluster analysis showed that, in sMPLC, unlike the high heterogeneity in the genome, the DNA methylation patterns were very similar among the multiple lesions from the same patient. **B** Based on the genome-wide DNA methylation patterns, the sMPLC and SPLC tumor samples could be directly clustered into two groups. **C** The DNA methylation level of SPLC tumors around the transcription starting site (TSS) was significantly higher than that in the sMPLC tumors. **D** and **E** DMP identified in sMPLC and SPLC tumors, compared to their normal lung tissues. The genes corresponding to the top 20 differences in DMP are listed at the two tips of the volcanic map. **F** DMP identified between sMPLC and SPLC tumors. The genes corresponding to the top 20 differences in DMP are listed at the two tips of the volcanic map. MC, multiple primary lung cancers; SC, single primary lung cancer

Genome-wide analyses of DNA methylation were conducted, and the density plot showed relatively similar methylation levels in the samples from the sMPLC and SPLC (Additional file 9: Fig. S1A). Principal component analysis (PCA) based on all CpG sites did not reveal any discernable separation between the two groups (Additional file 9: Fig. S1B). The heatmap and cluster analysis showed that, in sMPLC, unlike the high heterogeneity in the genome, the DNA methylation patterns were very similar among the multiple lesions from the same patient. Based on the genome-wide DNA methylation patterns, the sMPLC and SPLC tumor samples could be directly clustered into two groups, indicating that the DNA methylation patterns were different between the two groups (Fig. 3A and B).

A total of 3364 differentially methylated positions (DMP) were found in sMPLC tumors (1936 hypomethylated and 1428 hypermethylated), and 20,444 DMP were found in SPLCs (11,995 hypomethylated and 8449 hypermethylated), compared to their normal lung tissues, respectively. These substantially methylated sites appear to be bimodal distribution and randomly distributed on 22 chromosomes (Additional file 9: Fig. S1C and D). The distribution percentages of the DMP on different gene regions were slightly different between sMPLC and SPLC (Additional file 9: Fig. S1E and F). The genes corresponding to the top 20 differences in DMP are listed at the two tips of the volcano plot (Fig. 3D and E). GO and KEGG analyses revealed the DMP in sMPLC and SPLC involved in different biological processes, cellular functions, and pathways (Additional files 6 and 9: Table S6 and Fig. S1G, H).

The PPI network analysis identified 12 essential differentially methylated genes, and related epigenetic function modules were established as statistically significant in the sMPLC group. Among these genes, *SPINT2, LNX1, FOXA2, and SOSTDC1* were reported to be related to tumor progression, whereas *TYROBP, TREM2, IL23R, CSF2RB, TRAF1,* and *TGFBR1* were closely associated with immune response. The genes with up‐regulated or down‐regulated methylation levels had complex interactions in the signaling network (Additional file 7: Table S7).

In the SPLC, 15 essential differentially methylated genes and epigenetic function modules were identified. PPI network analysis revealed these genes and modules were mainly related to tumor progression (*LOXL1, IKBKE, LNX1, FOXA2, FGF17,* and *FGF2*) and immune response (*STAT4, IL23R, LTA, ITK, and IL19). LNX1, FOXA2,* and *IL23R* overlapped between sMPLC and SPLC, while other modules indicated that the DNA methylation patterns in sMPLC were different from SPLC. The enrichment results are presented in detail in Additional file 7: Table S7.

### DNA methylation discrepancies between sMPLC and SPLC

**Fig. 4:**
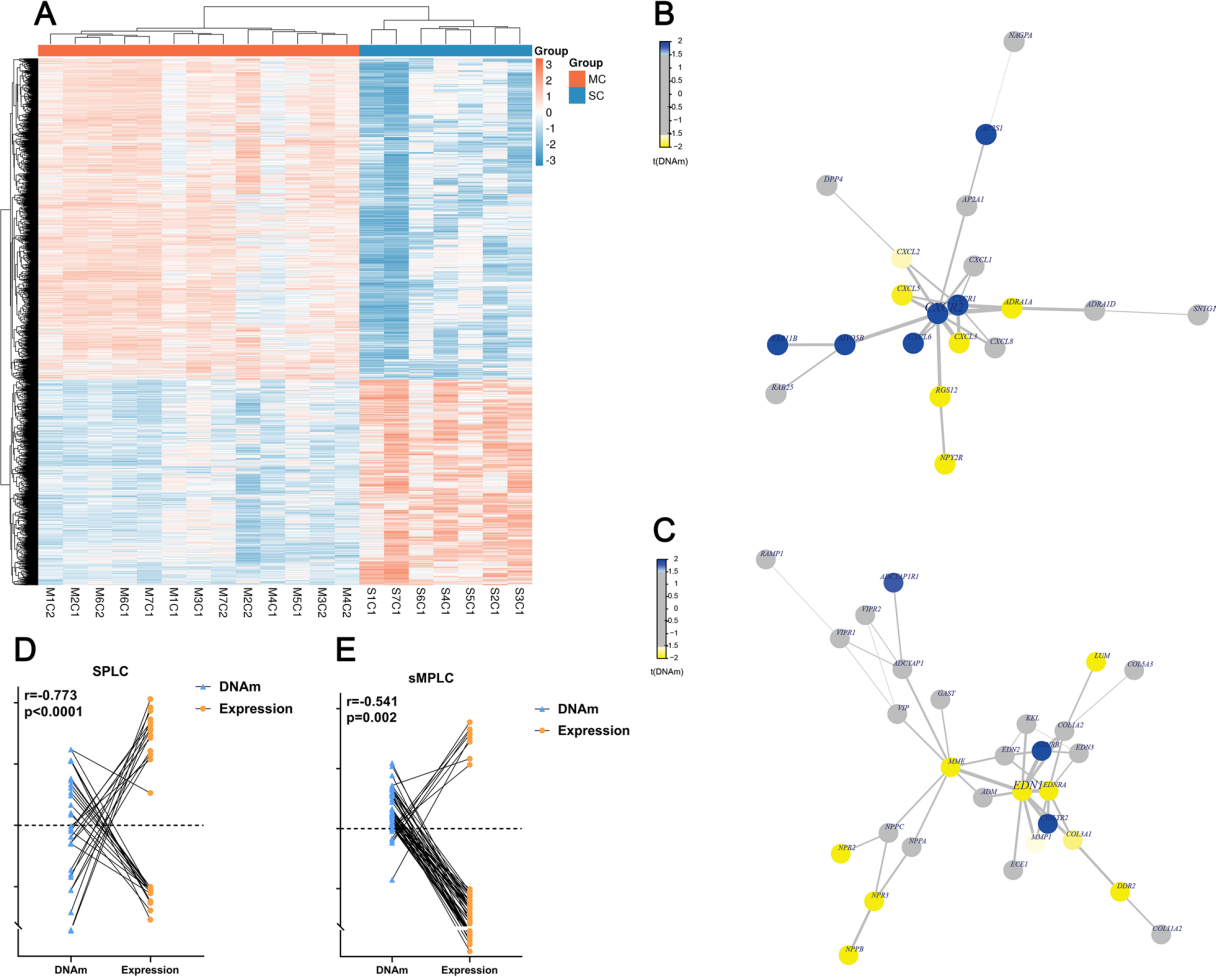
DNA methylation discrepancies between sMPLC and SPLC. **A** Heatmap generated from clustering analysis illustrated the DMP between sMPLC and SPLC. **B** and **C** The PPI network analysis identified CXCR2, EDN1, and ADRA1A as essential differentially methylated genes. The epigenetic function modules were established to illustrate the interactions of the related genes. The CXCR2 and ADRA1A closely interacted and were shown in a same module. **D** and **E** Aberrant DNA methylation at the TSS region was significantly related to the abnormal expression of immune-related genes. In sMPLC, the *r* = − 0.541, *p* = 0.002; in SPLC, the *r* = − 0.773, *p* < 0.0001

We next investigated the DNA methylation discrepancies between the two groups by directly comparing the DNA methylation status between the tumor samples from sMPLC and SPLC. Interestingly, the DNA methylation pattern around the transcription starting site (TSS) differed between the two groups. And the methylation level of SPLC tumors around TSS was significantly higher than that in the sMPLC (Fig. 3C, Additional file 9: Fig. S2B and C). A total of 8068 DMP (3201 hypomethylated and 4867 hypermethylated) were identified. Heatmap generated from clustering analysis illustrating DMP between sMPLC and SPLC, and the genes corresponding to the top 20 differences in DMP are listed at the two tips of the volcano plot (Figs. 3F and 4A). GO functional analysis showed that these DMP are involved in different biological processes, cellular components, and molecular functions (Additional file 9: Fig. S2A). The top 20 prominently enriched KEGG pathways of the DMP are presented in Table 1. The most significant pathway was the cholinergic synapse pathway (*p* = 3.08E-06). The pathway with the greatest number of DMPs was the chemokine signaling pathway (23 DMPs, *p* = 1.33E-05).

**Table 1:** Top 20 prominently enriched KEGG pathways of the DMP between sMPLC and SPLC

Pathway ID	Pathway description	Diff gene/all genes in this pathway	Rich factor	*p* value	*Q* value	Gene list
hsa04725	Cholinergic synapse	18/112	3.66004	3.08E-06	0.000831	CREB3L3, KCNQ5, GNG12, PIK3R3, ADCY5, BCL2, AKT3, CHRNA3, PLCB2, CAMK2B, ADCY7, KCNQ2, ADCY9, PRKCB, CHRM1, GNG7, GNG4, CACNA1A
hsa04973	Carbohydrate digestion and absorption	6/44	3.105489	0.007932	0.082373	LCT, PIK3R3, AKT3, PLCB2, PRKCB, GNAT3
hsa04713	Circadian entrainment	13/96	3.083923	0.000309	0.020872	CACNA1I, GNG12, PRKG1, ADCY5, GRIN2B, PLCB2, CAMK2B, ADCY7, ADCY9, RYR3, PRKCB, GNG7, GNG4
hsa04724	Glutamatergic synapse	15/114	2.996524	0.000172	0.015514	SLC1A7, DLGAP1, GRM4, GNG12, ADCY5 GRIN2B, PLCB2, ADCY7, ADCY9, PPP3R1, PRKCB, GRM7, GNG7, GNG4, CACNA1A
hsa04923	Regulation of lipolysis in adipocytes	7/54	2.952131	0.006607	0.081085	PRKG1, PIK3R3, ADCY5, AKT3, ADCY7, ADCY9, PTGER3
hsa04062	Chemokine signaling pathway	23/184	2.846698	1.33E-05	0.001798	CXCL2, NFKBIA, CXCR1, GNG12, TIAM1, PIK3R3, ADCY5, AKT3, PLCB2, PRKCZ, ADCY7, ADCY9, ROCK2, CCR7, STAT3, CCR8, PRKCB, PARD3, CCL1, GNG7, PREX1, GNG4, ELMO1
hsa04931	Insulin resistance	13/107	2.766884	0.000896	0.03457	NFKBIA, PRKCE, CREB3L3, PIK3R3, PRKAG2, AKT3, PCK1, PRKCZ, STAT3, PRKCB, GFPT2, RPS6KA2, PPP1R3E
hsa05143	African trypanosomiasis	4/34	2.679245	0.037482	0.206534	LAMA4, PLCB2, PRKCB, TLR9
hsa04911	Insulin secretion	10/85	2.679245	0.003709	0.066757	ABCC8, CREB3L3, ADCY5, PLCB2, KCNMB2, CAMK2B, ADCY7, ADCY9, PRKCB, KCNMB3
hsa04925	Aldosterone synthesis and secretion	11/95	2.636941	0.002906	0.07134	CACNA1I, PRKCE, CREB3L3, ADCY5, KCNK9, PLCB2, CAMK2B, ADCY7, ADCY9, PRKCB, SCARB1
hsa04728	Dopaminergic synapse	15/131	2.607662	0.000783	0.035218	CREB3L3, GNG12, LRTOMT, ADCY5, GRIN2B, AKT3, PLCB2, CAMK2B, PPP2R2B, PRKCB, GNG7, PPP2R5C, KIF5C, GNG4, CACNA1A
hsa04727	GABAergic synapse	10/88	2.587907	0.004817	0.081294	SLC6A11, GNG12 ADCY5, GABRG3, ADCY7, ADCY9, PRKCB, GNG7, GNG4, CACNA1A
hsa04213	Longevity regulating pathway—multiple species	7/62	2.571211	0.014668	0.11315	PIK3R3, ADCY5, PRKAG2, AKT3, ADCY7, ADCY9, HSPA8
hsa04071	Sphingolipid signaling pathway	13/118	2.508954	0.002244	0.060583	PRKCE, PIK3R3, BCL2, AKT3, PLCB2, PRKCZ, PPP2R2B, SGMS2, ROCK2, PRKCB, DEGS2, PPP2R5C, SGMS1
hsa05032	Morphine addiction	10/91	2.502592	0.006179	0.079448	GNG12, ADCY5, GABRG3, ADCY7, ADCY9, PRKCB, PDE8A, GNG7, GNG4, CACNA1A
hsa04512	ECM–receptor interaction	9/82	2.49954	0.008714	0.084029	LAMB3, LAMA4, SV2B, ITGB5, ITGB4, ITGA11, CD47, LAMA3, LAMB1
hsa04930	Type II diabetes mellitus	5/46	2.47539	0.035767	0.201192	ABCC8, PRKCE, PIK3R3, PRKCZ, CACNA1A
hsa04660	T cell receptor signaling pathway	11/103	2.43213	0.005554	0.078927	NFKBIA, PAK6, GRAP2, CBLB, PIK3R3, AKT3, CD247, PPP3R1, ZAP70, NCK2, MAP3K14
hsa05146	Amoebiasis	10/94	2.422722	0.007834	0.084603	LAMB3, LAMA4, PIK3R3, PLCB2, CD1D, ITGAM, PRKCB, IL1R2, LAMA3, LAMB1
hsa05223	Non-small cell lung cancer	7/66	2.41538	0.020755	0.136676	PIK3R3, AKT3, RARB, STAT3, PRKCB, EML4, ALK

The PPI network analysis identified three essential differentially methylated genes, *CXCR2, EDN1,* and *ADRA1A*, and related epigenetic function modules were established to illustrate the interactions of the related genes (Fig. 4B and C). The *CXCR2* and *ADRA1A* closely interacted, and the signaling network of related genes indicated that the function module might involve in the immune response regulation in the tumor microenvironment (Table 2).

**Table 2:** Essential differentially methylated genes and the genes in the epigenetic function modules

Entrez ID	Symbol	Full name	Function	Size	Genes in the module
148	ADRA1A	Adrenoceptor alpha 1A	Immune response	18	ADRA1A, NOS1, ADRA1D, CXCR1, CXCR2, CXCL3, CXCL6, CXCL5, CXCL2, CXCL1, CXCL8, MYO5B, RAB11B, RAB25, RASD1, DPP4, SNTG1, VAC14
1906	EDN1	Endothelin 1	Tumorigenesis	29	EDN1, EDNRB, KEL, EDNRA, COL1A2, CYSLTR2, MME, COL3A1, ECE1, ADM, MMP1, EDN2, EDN3, VIP, ADCYAP1, ADCYAP1R1, VIPR2, VIPR1, COL5A3, DDR2, COL11A2, LUM, NPPA, NPPC, NPR3, NPPB, NPR2, GAST, RAMP1
3579	CXCR2	C-X-C motif chemokine receptor 2	Immune response	20	CXCR2, RGS12, AP2A1, CXCL8, CXCR1, CXCL2, ADRA1A, CXCL1, CXCL5, MYO5B, CXCL3, CXCL6, NPY2R, RAB11B, AP2S1, RAB25, ADRA1D, DPP4, SNTG1, NAGPA

### Immune characterization of SPLC and sMPLC patients

**Fig. 5:**
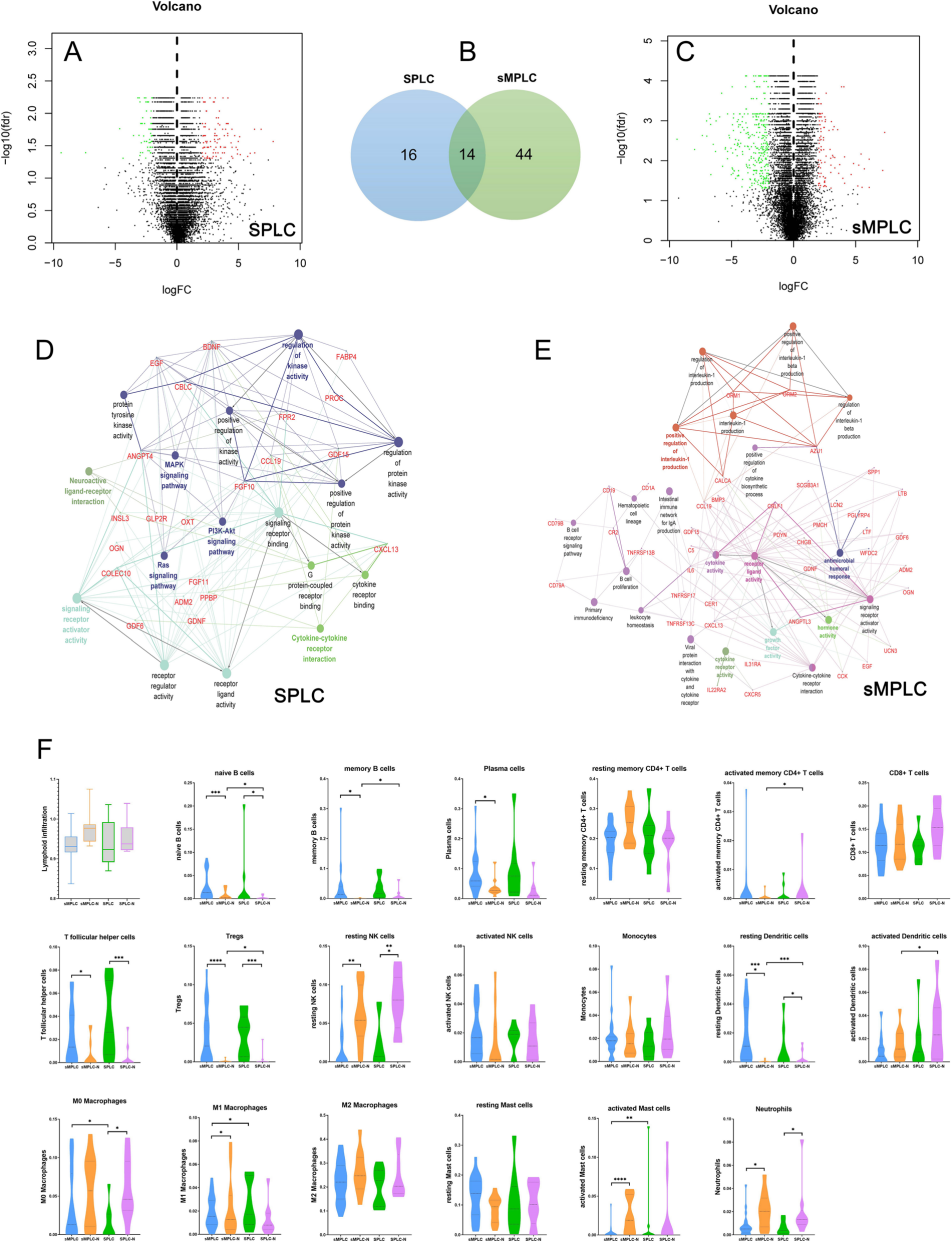
**A**, **B** and **C** In SPLC patients, 30 immune-related DEGs were identified with 15 genes down-regulated and 15 genes up-regulated. In sMPLC patients, 58 immune-related DEGs were identified with 50 genes down-regulated and 8 genes up-regulated. Fourteen DEGs were shared in both groups. **D** and **E** Visualizing biomolecular interaction network in SPLC and sMPLC. **F** CIBERSORT analyses revealed immune cell infiltrating differences between SPLC and sMPLC patients. In both groups, tumor tissues showed more significant infiltration of naïve B cells, TfH cells, Tregs, activated NK cells, resting dendritic cells, and macrophages. Moreover, in the sMPLC group, memory B cells, plasma cells, and activated mast cells were significantly more infiltrated in tumor tissues than normal lung tissues. Interestingly, a certain group of immune cells (B cells, dendritic cells, Tregs, and activated memory CD4^+^ T cells) showed significant infiltrating differences even in normal lung tissues between SPLC and sMPLC patients. *, *p* < 0.05; **, *p* < 0.01; ***, *p* < 0.001. sMPLC, tumors from sMPLC group; sMPLC-N, normal lung tissues from sMPLC group; SPLC, tumors from SPLC group; SPLC-N, normal lung tissues from SPLC group

In the SPLC group, 30 immune-related DEGs were identified with 15 genes down-regulated and 15 genes up-regulated (Fig. 5A and B, Additional file 8: Table S8). The significant GO terms and KEGG pathways analyses showed that these DEGs were related to multiple biological processes, molecular functions, and pathways involved in immune response regulation (Additional file 9: Fig. S3A and C). Interestingly, our data identified more immune-related DEGs in sMPLC patients (50 genes down-regulated and 8 genes up-regulated). Only 14 DEGs were shared between the two groups (Fig. 5B and C, Additional file 8: Table S8). Different from SPLC patients, more immune-related biological processes and pathways, such as B cell activation, B cell receptor signaling pathway, and IL-17 signaling pathway, were identified in the sMPLC group (Additional file 9: Fig. S3B and D). Visualizing biomolecular interaction networks [20] are shown in Fig. 5D and E. Moreover, we investigated the correlation between DNA methylation and gene expression. Aberrant DNA methylation at the TSS region was found in most of these abnormal expressed immune-related genes, indicating that DNA methylation may involve in the immune response regulation of early-stage lung cancer (Fig. 4B and C).

We then evaluated the TILs in tumor and non-tumor tissues from SPLC and sMPLC patients. In both groups, tumor tissues showed more significant infiltration of naïve B cells, TfH cells, Tregs, activated NK cells, resting dendritic cells, and macrophages. Moreover, in the sMPLC group, memory B cells, plasma cells, and activated mast cells were significantly more infiltrated in tumor tissues than in normal lung tissues (Fig. 5F). Immunofluorescence analyses showed similar results to the CIBERSORT (Additional file 9: Fig. S3E). Together, our data demonstrated the existence of immune profile discrepancies between SPLC and sMPLC patients. DNA methylation may involve immune dysregulation in the tumor microenvironment of SPLC and sMPLC.

## Discussion

The majority of early-stage sMPLC are AIS, MIA/IAC lesions that present as coexisting GGO, mGGO, and/or solid nodules [9, 11]. Previous studies have reported significant genetic heterogeneity among the coexisting lesions. With similar clinical characteristics to early-stage SPLC, there remains confusion about how these lesions occur synchronously. In this study, we investigated gene mutation status in a large cohort of sMPLC patients and compared the genetic mutation changes to SPLC. Several genetic details were demonstrated. Moreover, for the first time, we demonstrated the genome-wide DNA methylation patterns of sMPLC and compared its difference with SPLC. To better understand the potential mechanisms for the occurrence of sMPLC, immune microenvironment characteristics were also investigated. To the best of our knowledge, this is the first study to explore the genetic, epigenetic, and immune profile discrepancies between early-stage SPLC and sMPLC patients. Several interesting results were found.

First, our data demonstrated that the TMB level was relatively lower in sMPLC lesions than in SPLC. Previous studies have reported that IAC lesions had higher TMB than AIS/MIA [21]. And a notable increase in the TMB was specifically identified in tumors of the solid subtype, compared with GGO and mGGO subtypes [22]. Similar results can be found in our study, both in sMPLC and in SPLC patients. Moreover, when comparing the TMB levels between sMPLC and SPLC nodules, significantly lower TMB levels were found in sMPLC, especially in GGO/mGGO lesions. Interestingly, the IAC nodules from sMPLC patients also had lower TMB levels than SPLC patients. Previous studies have explored the feasibility and safety of neoadjuvant therapy with immune checkpoint inhibitors (ICIs) in early-stage NSCLC [23, 24], and TMB has been demonstrated to be a useful predictive biomarker [25, 26]. Our data suggested a cautious approach when applying immunotherapy to sMPLC patients due to its lower TMB levels. A further clinical study will be conducted to investigate the adjuvant therapy with ICIs in sMPLC patients.

Secondly, our data demonstrated that sMPLC had a similar gene mutational landscape to SPLC, despite some genetic discrepancies in subgroups. Consistent with previous studies, our data revealed a high proportion of patients harboring EGFR mutations in both sMPLC and SPLC patients, and nodules from sMPLC showed significant genetic heterogeneity [12, 27]. The EGFR mutation rate significantly increased from GGO lesion to mGGO lesion and solid lesion. Moreover, the EGFR mutation frequency was different between the two groups, especially in GGO (44.8% vs. 61.7%, *p* < 0.05) and solid lesions (79.2% vs. 41.3%, *p* < 0.01). Previous studies have shown that solid IAC nodules were less likely to harbor EGFR mutations than GGO and mGGO subtypes [22, 28]. Our study confirmed these findings in SPLC patients but not in sMPLC, indicating that unique genetic features exist in sMPLC.

Previous studies have described the effectiveness of EGFR-TKI treatment of SPLC patients [29]. However, only a few studies reported the potency of EGFR-TKI treatment in sMPLC, especially in GGO lesions [27, 30]. Given the genetic heterogeneity, coexisting lesions in sMPLC may have a different response to the TKI treatment [30]. Ye and colleagues reported lesions’ different responses to TKI treatment before surgery. The patient underwent surgical resection of the gefitinib-insensitive peripheral lesion and continued gefitinib treatment for the residual gefitinib-sensitive GGO lesions [31]. He and colleagues reported the changes of 134 lesions in 66 sMPLC patients treated with postoperative EGFR-TKIs. The response rate was 23.9%, and most of the responded lesions were larger and mGGO lesions [32]. Our data provided additional evidence for the previous studies, suggesting that EGFR-TKIs may have limited efficacy on multifocal lung cancer, especially for the GGO lesions.

Thirdly, our study for the first time investigated the genome-wide DNA methylation patterns in sMPLC. Unlike the high heterogeneity in genetic mutation, the DNA methylation patterns were very similar among the multiple lesions from the same patient, suggesting underlying correlations among the coexisting nodules at the DNA methylation level. The DMPs identified in SPLC and sMPLC and further functional analyses revealed that the DNA methylation patterns were different between the two groups. Several differentially methylated genes, such as STAT4, IL23R, CXCR2, and ITK, were identified, and some of these genes have key essential roles in regulating the immune response. The PPI network identified CXCR2 and ADRA1A as important methylated genes and closely interacted in sMPLC. ADRA1A belongs to the G-protein-coupled receptor superfamily, which activates mitogenic responses and regulates cells' growth and proliferation. CXCR2 gene encoded protein that belongs to the G-protein-coupled receptor family, functioning as a receptor for IL8. Promoter aberrant hypermethylation of ADRA1A might contribute to the initiation of hepatocellular carcinoma. Knockdown ADRA1A might result in the abnormal expression of genes enriched in cytokine–cytokine receptor interaction pathways [33]. CXCR2 in NSCLC has been studied mainly in stromal cells and was known to increase tumor inflammation and angiogenesis. Promoter hypermethylation of the CXCR2 axis has been determined in NSCLC and may be an important therapeutic target in lung adenocarcinoma [34]. The CXCR2-ADRA1A function module generated from our data included several immune-related genes, such as CXCL5, CXCL13, RGS12, and CXCR1, indicating that the DNA methylation function module might involve in the immune response regulation in the tumor microenvironment of sMPLC.

Fourthly, our data identified several immune-related genes and pathways that were abnormally regulated in sMPLC. Further analyses showed different immune profiles between sMPLC and SPLC. Moreover, the TILs were different between the two groups. It has been well accepted that antitumor immune surveillance plays a critical role during carcinogenesis. Previous studies have demonstrated that the immunoediting had started before the tumor invasion [35]. Abnormally infiltrated B cells, Tregs, macrophages, and NK cells could be found in the early stage of cancer and play a critical role in tumor initiation and development [35–37]. Our data confirmed these results and revealed the immune response discrepancies between SPLC and sMPLC. Moreover, when integrated with DNA methylation data, the abnormal expression of several immune-related genes was closely correlated with aberrant DNA methylation. Previous studies have demonstrated that DNA methylation underlies the specification of immune cell lineages. Both innate and adaptive immune lineages are specified by distinct epigenetic mechanisms via combinatorial and context-dependent use of crucial transcription factors [38]. Our data showed that DNA methylation might involve in the immune dysregulation in the tumor microenvironment of early-stage lung cancer.

The concept of ‘field cancerization’ hypothesized that multiple primary cancers might arise simultaneously and coexist with subclinical precursor lesions within a defined field [39]. It was proposed to explain the development of multiple primary tumors and locally recurrent cancer. The development of an expanding pre-neoplastic field appeared to be a critical step in epithelial carcinogenesis [40]. Our study showed that the DNA methylation patterns in sMPLC were very similar among the multiple lesions from the same patient, indicating that DNA methylation may involve in the formation of a pre-neoplastic field. In addition, the aberrant DNA methylation was closely related to the abnormal expression of immune-related genes, suggesting that DNA methylation may contribute to the immune response regulation in the cancer field of sMPLC. DNA methylation in the cancer field of the lung might play a critical role in the initiation and development of sMPLC, which potentially lead to the conclusion that the occurrence of sMPLC has its unique molecular regulation mechanisms; novel and specific epigenetic and immunotherapies should be explored in the future.

There are some limitations to this study. First, this study used a single-institution Chinese database and enrolled only surgically resected cases. As this was a retrospective study, a validation study was required to confirm our conclusion. Second, the sample size might be small. A total of 486 lung nodules were included for gene mutational analysis, which is the largest genetic study for sMPLC, and several genetic details were demonstrated. However, DNA methylation and RNA-seq analyses were only performed in a small number of patients. Due to the relatively insufficient amount of tumor samples of early-stage sMPLC, it is difficult to carry out multi-omics analyses on the same sample at the same time. The present study provides new clues and information for understanding the occurrence and development of sMPLC from DNA methylation and immune response aspect; a larger cohort including more sMPLC patients is needed for further investigation. Third, with the current wide panel-genomic sequencing strategy, only 1021 cancer-related genes were investigated. Some important genomic information might be lost. Therefore, future studies with WGS technologies may help further investigate the biology of sMPLC.

## Conclusion

Our study for the first time demonstrated genetic, DNA methylation, and immune profile discrepancies between sMPLC and SPLC. Relative to the similar genetic mutational landscape, the DNA methylation patterns and related immune profiles were significantly different between sMPLC and SPLC, indicating their essential roles in the initiation and development of sMPLC. Our study provided new insight into understanding molecular and immunopathological mechanisms of the pathogenesis of sMPLC.

## Materials and methods

### Patients and samples

From June 2017 to October 2019, 337 patients with early-stage primary non-small cell lung cancer that underwent surgical resection were enrolled in this study, including 137 sMPLC patients and 200 SPLC patients. Patients who received preoperative induction therapy, such as chemotherapy or radiotherapy, or a history of malignancy were excluded. All patients were staged postoperatively according to the revised TNM guidelines classification criteria. For sMPLC patients, the TNM stage was determined by the highest stage of all lesions according to the 8th TNM classification guidelines [41, 42]. Demographic variables were collected from all patients in two groups including age, gender, smoking status, stage, histological types, tumor size, and CT features. This study was approved by the Institutional Review Board of Second Xiangya Hospital, and written informed consent was signed by all participants for their clinical records.

### CT evaluation

All the patients received a high-resolution CT scan before the surgery. The ground-glass opacification (GGO) lesion, mixed GGO (mGGO) lesion, and solid lesion were defined according to previous studies [1, 43, 44]. All nodules were subsequently evaluated to estimate the extent of the GGO lesion in a thin-section CT scan with 1-mm collimation. The size of the nodules was determined preoperatively based on a thin-section CT scan.

For patients with SPLC, all the patients received a high-resolution CT scan every 6 months after curative surgical resection. No new lung lesions were detected during the follow-up period so the diagnosis stayed SPLC instead of re-diagnosing as MPLC according to the current diagnostic criteria [5, 9].

### Tissue samples and gene mutation analysis

Fresh tissues were obtained from each nodule and healthy lung tissue 5 cm from each lesion. Healthy tissues were pathologically confirmed postoperatively. Genomic DNA was extracted from fresh tissues using the QIAamp DNA Tissue Kit (Qiagen, Germany). All the samples were subjected to wide panel-genomic sequencing (pan-cancer 1021-gene panel, Geneplus Technology Inc.) at the coverage depth of 1800 × . DNA from peripheral blood mononuclear cells (PBMCs) of the same patients served as a germline DNA reference. The detection and data analysis were performed as described previously [45, 46].

### Infinium human methylation EPIC array using 850 K BeadChip and data analysis

500 ng DNA of each sample was bisulfite-converted using the EZ DNA Methylation Gold Kit (Zymo Research, USA) following the manufacturer’s protocol. Genome-wide DNA methylation was assessed using the Infinium Human Methylation 850 K BeadChip (Illumina Inc., USA) according to the manufacturer’s instructions.

The array data were analyzed using the ChAMP package in R to derive the methylation level. We used the *β* value to represent the proportion of methylated each CpG site. First, we filtered probes with detection *p* value < 0.01, probes with < 3 beads in at least 10% of samples, non-CpG probes, multi-hit probes, probes located in chromosomes X and Y, and SNP-related probes, the final probes for subsequent analysis were 820,000. Then, *β* value matrix was normalized using BMIQ to adjust type I and type II probe bias. Next, we used singular value decomposition analysis to analyze the batch effect caused by BeadChip Slide and Array and then applied Comba to correct this batch effect [47]. We annotated all CpG sites using EPICanno.ilm10b4.hg19. Differentially methylated CpGs positions (probes) were calculated by the champ.DMP function; the adjusted *p* values were computed using the Benjamini–Hochberg method. CpGs with |Δ*β*| ≥ 0.20 and adjusted *p* value ≤ 0.05 were considered as DMP. Differentially methylated regions were calculated using ProbeLasso [48]. All CpGs were for DMR calling, and minSigProbesLasso, lassoRadius, and boundary were the default values. To evaluate the DMP and DMR biologic processes, cellular components, molecular and pathways, we conducted Gene Ontology (GO) and KEGG enrichment analysis using the R package. Finally, we use functional epigenetic modules to infer differentially methylated gene modules in protein-to-protein interaction network (PPI) using the FEM package.

### RNA extraction

The fresh tissue samples were washed with distilled deionized water and preserved using liquid nitrogen for quick freezing. The RNA extraction of tumor specimens was performed using the RNeasy Mini Kit (Qiagen), according to the manufacturer’s recommendations. RNA quantity and purity were measured with the NanoDrop ND-1000 spectrophotometer (Thermo Scientific). RNA integrity, determined by the RNA integrity number (RIN), was determined with the 2100 Bioanalyzer (Agilent).

### RNA-seq and DEGs analysis

The differentially expressed genes (DEGs) between tumor samples and normal lung tissues were identified with |log FC| > 2, which was defined as multiple differences in the gene expression greater than two between the tumor samples and normal lung tissues and false discovery rate *p* value (FDR-P) < 0.05 using the ‘limma’ R package. We further analyzed the immune-related DEGs between these two groups using the ‘VennDiagram’ package, based on 1769 immune-related genes in the Immunology Database and Analysis Portal (https://www.immport.org/shared/home).

### GO and KEGG analyses

The gene ontology (GO) and Kyoto Encyclopedia of Genes and Genomes (KEGG) enrichment pathway analyses were utilized to explore the molecular function of the DEGs by clusterProfiler, org.Hs.eg.db, enrichplot, ggplot2’ R packages. A *p* value < 0.05 and FDR q value < 0.25 were considered statistically significant. We imported data of these immune-related DEGs into Cytoscape software (version 3.8.2) and analyzed the data using the ClueGO plugin. The GO and KEGG datasets were selected to perform the function network analysis, and the GO terms included the cellular component (CC), molecular function (MF), biological process (BP), and adjusted *p* < 0.05 as a statistically significant value.

### Estimation of tumor-infiltrating lymphocytes (TILs)

CIBERSORT (http://cibersort.stanford.edu/) was used to quantify the abundance of 22 infiltrating immune cell types in each sample, with the algorithm run using the LM22 signature matrix at 1000 permutations. These tumor-infiltrating immune cells included B cells, T cells, natural killer cells, macrophages, dendritic cells, eosinophils, and neutrophils. For each tumor sample, the sum of all evaluated immune cell type fractions equaled 1 [49, 50]. Immunofluorescence analysis was used to validate the infiltration of TILs using the expression of CD3, CD4, CD8, and CD68 (Abcam, USA), respectively.

### Statistical analysis

The descriptive statistics for categorical variables were described as frequencies and numbers (percentages). Nominal categorical variables were compared using the Pearson’s chi-square test or Fisher’s exact test, when appropriate. Continuous variables were compared by using the t test. The logistic regression model was used for multivariable analysis to determine the relationship between clinical confounders and gene mutation status. All analysis was performed with SPSS software for Windows (Version 24.0, Chicago, IL, USA), R (version 4.0.5), and PRISM software (version 8.0, GraphPad Software, La Jolla, CA). A *p* value less than 0.05 was considered statistically significant.

## Supplementary Information

**Additional file 1: Table S1.** (**A**) Clinical characteristics of all the patients. (**B**) Clinical characteristics of the patient for genome-wide DNA methylation and RNA-seq analyses.

**Additional file 2: Table S2.** Tumor mutation burden in SPLC and sMPLC patients.

**Additional file 3: Table S3.** (**A**) Gene mutation landscape in SPLC patients—gender-oriented. (**B**) Gene mutation landscape in SPLC patients—age-oriented. (**C**) Gene mutation landscape in SPLC patients—nodule size-oriented. (**D**) Gene mutation landscape in SPLC patients—CT feature-oriented. (**E**) Gene mutation landscape in SPLC patients—histology-oriented. (**F**) Gene mutation landscape in sMPLC patients—gender-oriented. (**G**) Gene mutation landscape in sMPLC patients—age-oriented. (**H**) Gene mutation landscape in sMPLC patients—nodule size-oriented. (**I**) Gene mutation landscape in sMPLC patients—CT feature-oriented. (**J**) Gene mutation landscape in sMPLC patients—histology-oriented.

**Additional file 4: Table S4.** (**A**–**F**) Genetical discrepancies between SPLC and sMPLc patients.

**Additional file 5: Table S5.** (**A**–**I**) Genetical discrepancies between SPLC and sMPLc patients.

**Additional file 6: Table S6.** (**A**) KEGG analysis results in sMPLC. (**B**) KEGG analysis results in SPLC.

**Additional file 7: Table S7.** Essential differentially methylated genes in epigenetic function modules and related genes in signaling network.

**Additional file 8: Table S8.** Immune-related DEGs in SPLC and sMPLC patients.

**Additional file 9: Fig. S1.** Genome-wide DNA methylation patterns in sMPLC and SPLC. **A** The density plot showed relatively similar methylation levels in the samples from the sMPLC and SPLC. **B** Principal component analysis (PCA) based on all CpG sites did not reveal any discernable separation between the two groups. **C** and **D** The substantially methylated sites appear to be bimodal distribution and randomly distributed on 22 chromosomes in sMPLC and SPLC. **E** and **F** The distribution percentages of the DMP on different gene regions were slightly different between sMPLC and SPLC. **G** and **H** GO analyses revealed the DMP in sMPLC and SPLC involved in different biological processes, cellular components, and molecular functions. **Fig. S2.** DNA methylation discrepancies between sMPLC and SPLC. **A** GO Functional Analysis showed that these DMP are involved in different biological processes, cellular components, and molecular functions. **B** and **C** DNA methylation level around the transcription starting site (TSS). The methylation patterns around the TSS region were different between sMPLC and SPLC. MC, sMPLC tumors; MN, paired normal lung tissues from sMPLC; SC, SPLC tumors; SN, paired normal lung tissues from SPLC. **Fig. S3.** RNA-seq and immunofluorescence analyses in sMPLC and SPLC. **A** and **B** KEGG pathways analyses in SPLC and sMPLC based on DEG. **C** and **D** Significant GO terms analyses in SPLC and sMPLC. More immune-related biological processes and pathways, such as B cell activation, B cell receptor signaling pathway, and IL-17 signaling pathway, were identified in the sMPLC group. **E** Immunofluorescence analyses of CD3, CD4, CD8, and CD68 confirmed CIBERSORT analysis results. The infiltration of CD3+, CD4+, and CD8+ T cells had no differences between sMPLC and SPLC. The number of CD68+ lymphocytes was significantly higher in sMPLC (*p* < 0.01). sMPLC, sMPLC tumors; sMPLC-N, paired normal lung tissues from sMPLC; SPLC, SPLC tumors; SPLC-N, paired normal lung tissues from SPLC.

## Data Availability

The datasets used and/or analyzed during the current study are available from the corresponding author upon reasonable request.
