# Supplementary material for: Genetic, DNA methylation, and immune profile discrepancies between early-stage single primary lung cancer and synchronous multiple primary lung cancer
Source: Clin Epigenetics. 2023 Jan 7;15:4. doi: 10.1186/s13148-023-01422-y (PMC9824942; doi:10.1186/s13148-023-01422-y)
Supplement: Supplementary file 8 — Additional file 8: Table S8. Immune-related DEGs in SPLC and sMPLC patients. [file 13148_2023_1422_MOESM8_ESM.docx]

Supplemental Table S8. Immune-related DEGs in SPLC and sMPLC patients.

| Immune-related DEGs in SPLC | Immune-related DEGs in sMPLC |
| --- | --- |
| ADM2, ANGPT4, ANGPTL7, BDNF, CAMP, CBLC, CCL19, CD1A, COLEC10, CXCL13, EGF, FABP4, FGF10, FGF11, FPR2, GDF15, GDF6, GDNF, GLP2R, IL22RA2, INSL3, NCR1, OGN, OXT, PPBP, PROC, RBP2, SERPIND1, SH2D1B, WFDC2 | CCK, GDF6, GLP2R, CER1, IL6, CALCA, OGN, FCGR3B, TNFRSF17, EGF, TNFRSF13B, CD79B, TSHR, CBLC, BMP3, C5, ORM1, LTB, GDNF, MUC4, PROC, HNF4G, ORM2, CD79A, CCL19, ADM2, S100B, IL31RA, SCTR, WFDC2, APOH, LTF, PDYN, ANGPTL3, TNFRSF13C, S100G, NR2E1, PGLYRP4, LCN2, PMCH, CHGB, CD1A, CXCR5, SPP1, AZU1, SLC10A2, GDF15, UCN3, CST4, IL22RA2, CD19, MMP12, CRLF1, HTR3A, SCGB3A1, CXCL13, CR2, MUC5AC |
