# Supplementary material for: Genetic, DNA methylation, and immune profile discrepancies between early-stage single primary lung cancer and synchronous multiple primary lung cancer
Source: Clin Epigenetics. 2023 Jan 7;15:4. doi: 10.1186/s13148-023-01422-y (PMC9824942; doi:10.1186/s13148-023-01422-y)
Supplement: Supplementary file 9 — Additional file 9: Fig. S1. Genome-wide DNA methylation patterns in sMPLC and SPLC. A The density plot showed relatively similar methylation levels in the samples from the sMPLC and SPLC. B Principal component analysis (PCA) based on all CpG sites did not reveal any discernable separation between the two groups. C and D The substantially methylated sites appear to be bimodal distribution and randomly distributed on 22 chromosomes in sMPLC and SPLC. E and F The distribution percentages of the DMP on different gene regions were slightly different between sMPLC and SPLC. G and H GO analyses revealed the DMP in sMPLC and SPLC involved in different biological processes, cellular components, and molecular functions. Fig. S2. DNA methylation discrepancies between sMPLC and SPLC. A GO Functional Analysis showed that these DMP are involved in different biological processes, cellular components, and molecular functions. B and C DNA methylation level around the transcription starting site (TSS). The methylation patterns around the TSS region were different between sMPLC and SPLC. MC, sMPLC tumors; MN, paired normal lung tissues from sMPLC; SC, SPLC tumors; SN, paired normal lung tissues from SPLC. Fig. S3. RNA-seq and immunofluorescence analyses in sMPLC and SPLC. A and B KEGG pathways analyses in SPLC and sMPLC based on DEG. C and D Significant GO terms analyses in SPLC and sMPLC. More immune-related biological processes and pathways, such as B cell activation, B cell receptor signaling pathway, and IL-17 signaling pathway, were identified in the sMPLC group. E Immunofluorescence analyses of CD3, CD4, CD8, and CD68 confirmed CIBERSORT analysis results. The infiltration of CD3+, CD4+, and CD8+ T cells had no differences between sMPLC and SPLC. The number of CD68+ lymphocytes was significantly higher in sMPLC (p < 0.01). sMPLC, sMPLC tumors; sMPLC-N, paired normal lung tissues from sMPLC; SPLC, SPLC tumors; SPLC-N, paired normal lung tissues from SPLC. [file 13148_2023_1422_MOESM9_ESM.pdf]

Supplemental Figures

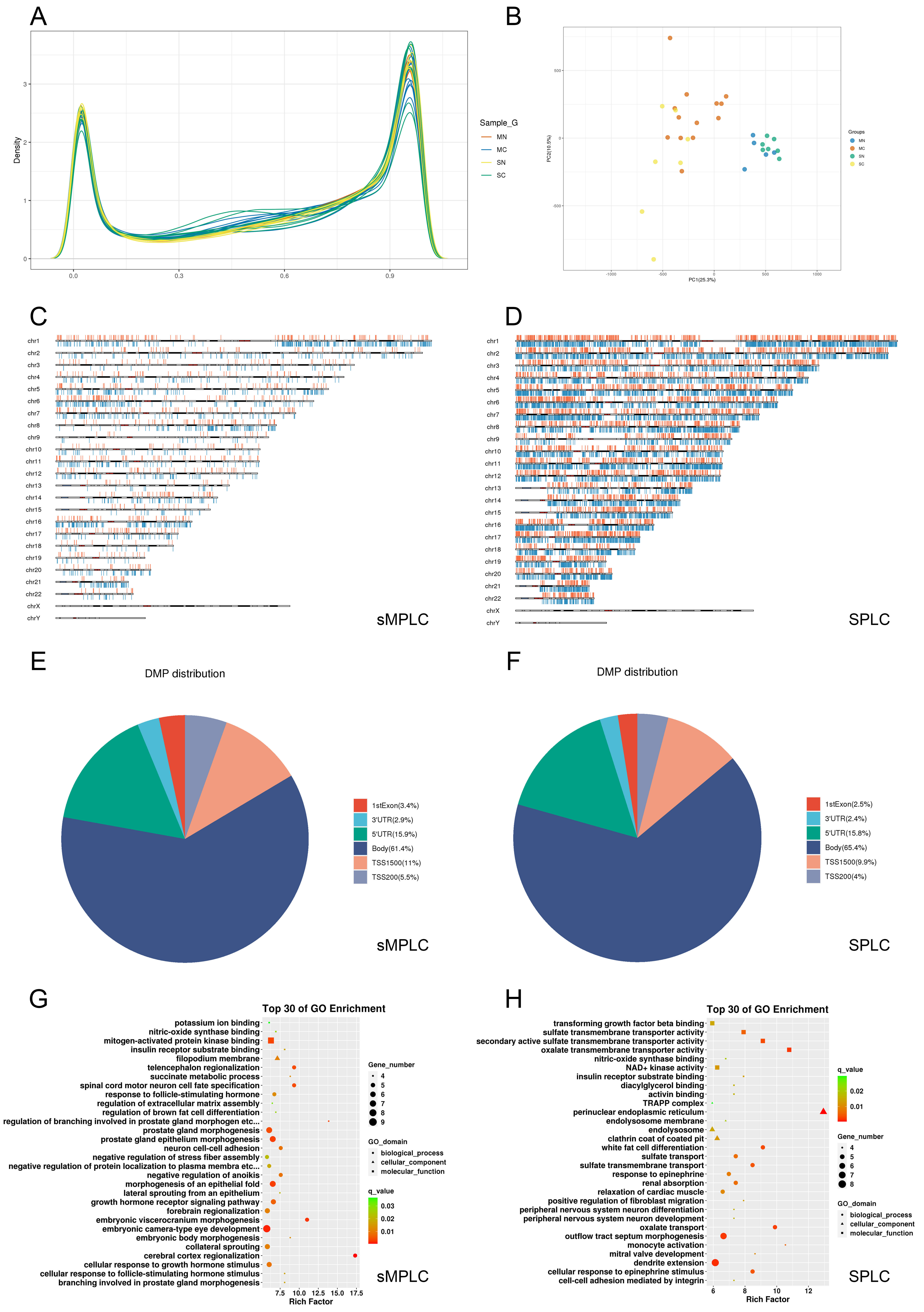

A
Density
Sample_G
MN
MC
SN
SC
B
PC1(25.3%)
PC2(10.5%)
Groups
MN
MC
SN
SC
C
sMPLC
D
SPLC
E
DMP distribution
1stExon(3.4%)
3'UTR(2.9%)
5'UTR(15.9%)
Body(61.4%)
TSS1500(11%)
TSS200(5.5%)
sMPLC
F
DMP distribution
1stExon(2.5%)
3'UTR(2.4%)
5'UTR(15.8%)
Body(65.4%)
TSS1500(9.9%)
TSS200(4%)
SPLC
G
Top 30 of GO Enrichment
potassium ion binding
nitric-oxide synthase binding
mitogen-activated protein kinase binding
insulin receptor substrate binding
filopodium membrane
telencephalon regionalization
succinate metabolic process
spinal cord motor neuron cell fate specification
response to follicle-stimulating hormone
regulation of extracellular matrix assembly
regulation of brown fat cell differentiation
regulation of branching involved in prostate gland morphogen etc...
prostate gland morphogenesis
prostate gland epithelium morphogenesis
neuron cell-cell adhesion
negative regulation of stress fiber assembly
negative regulation of protein localization to plasma membra etc...
negative regulation of anoikis
morphogenesis of an epithelial fold
lateral sprouting from an epithelium
growth hormone receptor signaling pathway
forebrain regionalization
embryonic viscerocranium morphogenesis
embryonic camera-type eye development
embryonic body morphogenesis
collateral sprouting
cerebral cortex regionalization
cellular response to growth hormone stimulus
cellular response to follicle-stimulating hormone stimulus
branching involved in prostate gland morphogenesis
Rich Factor
Gene_number
GO_domain
biological_process
cellular_component
molecular_function
q_value
sMPLC
H
Top 30 of GO Enrichment
transforming growth factor beta binding
sulfate transmembrane transporter activity
secondary active sulfate transmembrane transporter activity
oxalate transmembrane transporter activity
nitric-oxide synthase binding
NAD+ kinase activity
insulin receptor substrate binding
diacylglycerol binding
activin binding
TRAPP complex
perinuclear endoplasmic reticulum
endolysosome membrane
endolysosome
clathrin coat of coated pit
white fat cell differentiation
sulfate transport
sulfate transmembrane transport
response to epinephrine
renal absorption
relaxation of cardiac muscle
positive regulation of fibroblast migration
peripheral nervous system neuron differentiation
peripheral nervous system neuron development
oxalate transport
outflow tract septum morphogenesis
monocyte activation
mitral valve development
dendrite extension
cellular response to epinephrine stimulus
cell-cell adhesion mediated by integrin
Rich Factor
q_value
Gene_number
GO_domain
biological_process
cellular_component
molecular_function
SPLC

Supplemental Figure S1. Genome-wide DNA methylation patterns in sMPLC and SPLC. **A)** The density plot showed relatively similar methylation levels in the samples from the sMPLC and SPLC. **B)** Principal component analysis (PCA) based on all CpG sites did not reveal any discernable separation between the two groups. **C) and D)** The substantially methylated sites appear to be bimodal distribution and randomly distributed on 22 chromosomes in sMPLC and SPLC. **E) and F)** The distribution percentages of the DMP on different gene regions were slightly different between sMPLC and SPLC. **G) and H)** GO analyses revealed the DMP in sMPLC and SPLC involved in different biological processes, cellular components, and molecular functions.

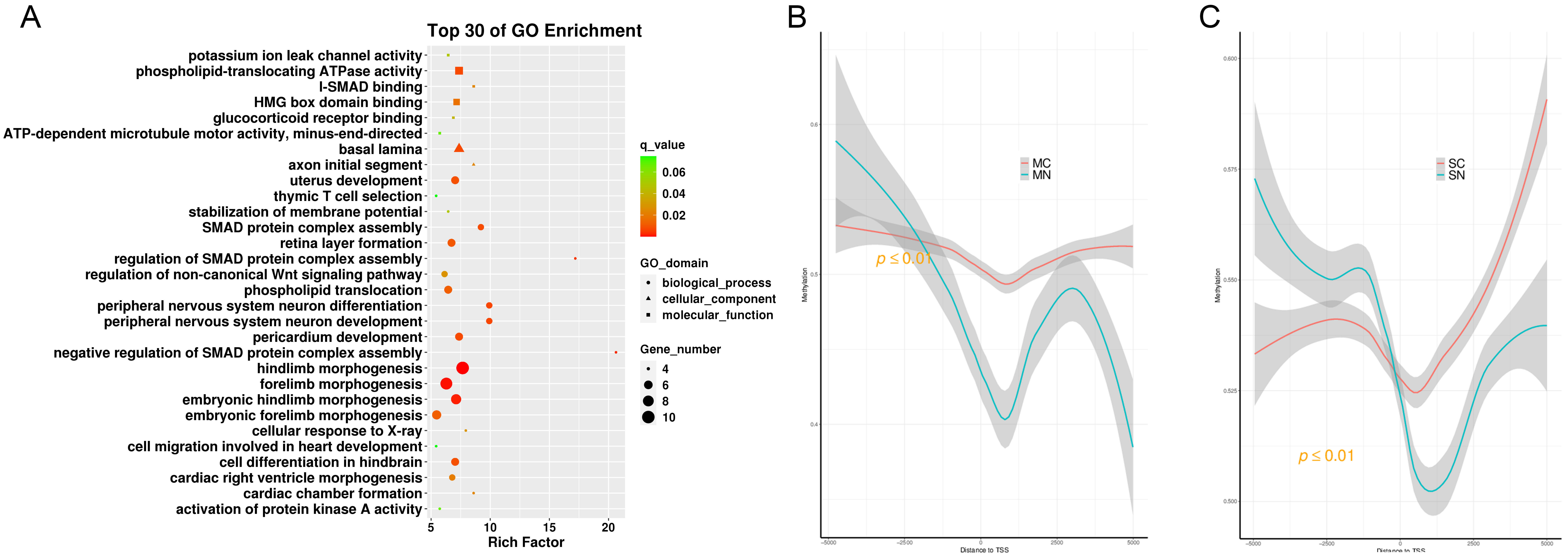
A
Top 30 of GO Enrichment
potassium ion leak channel activity
phospholipid-translocating ATPase activity
I-SMAD binding
HMG box domain binding
glucocorticoid receptor binding
ATP-dependent microtubule motor activity, minus-end-directed
basal lamina
axon initial segment
uterus development
thymic T cell selection
stabilization of membrane potential
SMAD protein complex assembly
retina layer formation
regulation of SMAD protein complex assembly
regulation of non-canonical Wnt signaling pathway
phospholipid translocation
peripheral nervous system neuron differentiation
peripheral nervous system neuron development
pericardium development
negative regulation of SMAD protein complex assembly
hindlimb morphogenesis
forelimb morphogenesis
embryonic hindlimb morphogenesis
embryonic forelimb morphogenesis
cellular response to X-ray
cell migration involved in heart development
cell differentiation in hindbrain
cardiac right ventricle morphogenesis
cardiac chamber formation
activation of protein kinase A activity
5
10
15
20
Rich Factor
q_value
0.06
0.04
0.02
GO_domain
biological_process
cellular_component
molecular_function
Gene_number
4
6
8
10
B
MC
MN
$p \leq 0.01$
Methylation
0.6
0.5
0.4
-5000
-2500
0
2500
5000
Distance to TSS
C
SC
SN
$p \leq 0.01$
Methylation
0.600
0.575
0.550
0.525
0.500
-5000
-2500
0
2500
5000
Distance to TSS

Supplemental Figure S2. DNA methylation discrepancies between sMPLC and SPLC. **A)** GO Functional Analysis showed that these DMP are involved in different biological processes, cellular components, and molecular functions. **B) and C)** DNA methylation level around the transcription starting site (TSS). The methylation patterns around the TSS region were different between sMPLC and SPLC. MC, sMPLC tumors; MN, paired normal lung tissues from sMPLC; SC, SPLC tumors; SN, paired normal lung tissues from SPLC.

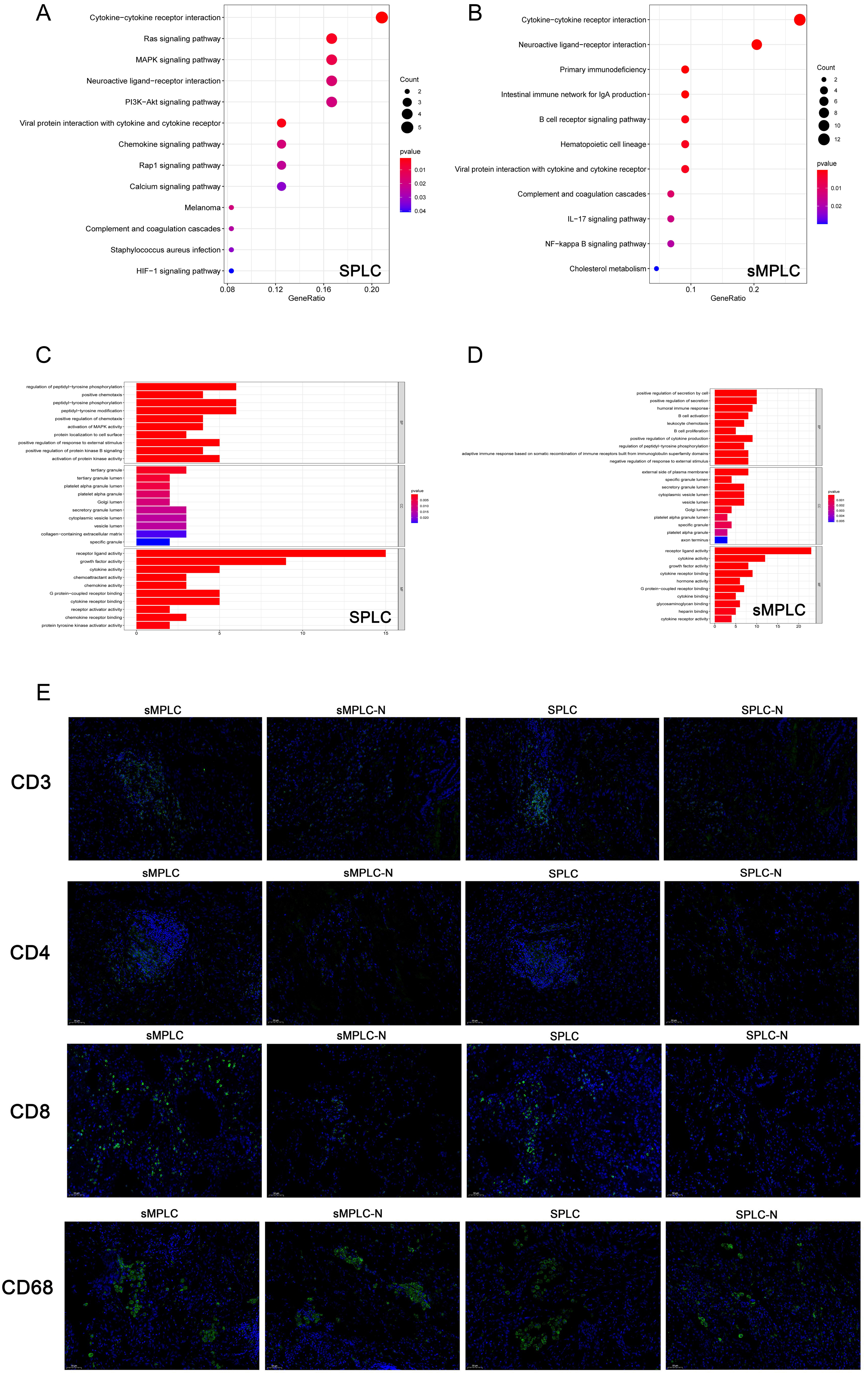
A
Cytokine−cytokine receptor interaction
Ras signaling pathway
MAPK signaling pathway
Neuroactive ligand−receptor interaction
PI3K−Akt signaling pathway
Viral protein interaction with cytokine and cytokine receptor
Chemokine signaling pathway
Rap1 signaling pathway
Calcium signaling pathway
Melanoma
Complement and coagulation cascades
Staphylococcus aureus infection
HIF−1 signaling pathway
SPLC
0.08
0.12
0.16
0.20
GeneRatio
Count
2
3
4
5
pvalue
0.01
0.02
0.03
0.04
B
Cytokine−cytokine receptor interaction
Neuroactive ligand−receptor interaction
Primary immunodeficiency
Intestinal immune network for IgA production
B cell receptor signaling pathway
Hematopoietic cell lineage
Viral protein interaction with cytokine and cytokine receptor
Complement and coagulation cascades
IL−17 signaling pathway
NF−kappa B signaling pathway
Cholesterol metabolism
sMPLC
0.1
0.2
GeneRatio
Count
2
4
6
8
10
12
pvalue
0.01
0.02
C
regulation of peptidyl−tyrosine phosphorylation
positive chemotaxis
peptidyl−tyrosine phosphorylation
peptidyl−tyrosine modification
positive regulation of chemotaxis
activation of MAPK activity
protein localization to cell surface
positive regulation of response to external stimulus
positive regulation of protein kinase B signaling
activation of protein kinase activity
tertiary granule
tertiary granule lumen
platelet alpha granule lumen
platelet alpha granule
Golgi lumen
secretory granule lumen
cytoplasmic vesicle lumen
vesicle lumen
collagen−containing extracellular matrix
specific granule
receptor ligand activity
growth factor activity
cytokine activity
chemoattractant activity
chemokine activity
G protein−coupled receptor binding
cytokine receptor binding
receptor activator activity
chemokine receptor binding
protein tyrosine kinase activator activity
SPLC
0
5
10
15
BP
CC
MF
pvalue
0.005
0.010
0.015
0.020
D
positive regulation of secretion by cell
positive regulation of secretion
humoral immune response
B cell activation
leukocyte chemotaxis
B cell proliferation
positive regulation of cytokine production
regulation of peptidyl−tyrosine phosphorylation
adaptive immune response based on somatic recombination of immune receptors built from immunoglobulin superfamily domains
negative regulation of response to external stimulus
external side of plasma membrane
specific granule lumen
secretory granule lumen
cytoplasmic vesicle lumen
vesicle lumen
Golgi lumen
platelet alpha granule lumen
specific granule
platelet alpha granule
axon terminus
receptor ligand activity
cytokine activity
growth factor activity
cytokine receptor binding
hormone activity
G protein−coupled receptor binding
cytokine binding
glycosaminoglycan binding
heparin binding
cytokine receptor activity
sMPLC
0
5
10
15
20
BP
CC
MF
pvalue
0.001
0.002
0.003
0.004
0.005
E
sMPLC
sMPLC-N
SPLC
SPLC-N
CD3
CD4
CD8
CD68

Supplemental Figure S3. RNA-seq and Immunofluorescence analyses in sMPLC and SPLC. **A) and B)** KEGG pathways analyses in SPLC and sMPLC based on DEG. **C) and D)** Significant GO terms analyses in SPLC and sMPLC. More immune-related biological processes and pathways, such as B cell activation, B cell receptor signaling pathway, and IL-17 signaling pathway, were identified in the sMPLC group. **E)** Immunofluorescence analyses of CD3, CD4, CD8, and CD68 confirmed CIBERSORT analysis results. The infiltration of $CD3^+$, $CD4^+$, and $CD8^+$ T cells had no differences between sMPLC and SPLC. The number of $CD68^+$ lymphocytes was significantly higher in sMPLC($p<0.01$). sMPLC, sMPLC tumors; sMPLC-N, paired normal lung tissues from sMPLC; SPLC, SPLC tumors; SPLC-N, paired normal lung tissues from SPLC.
